# Multi-omics analyses of gut microbiota via 16S rRNA gene sequencing, LC-MS/MS and diffusion tension imaging reveal aberrant microbiota-gut-brain axis in very low or extremely low birth weight infants with white matter injury

**DOI:** 10.1186/s12866-023-03103-5

**Published:** 2023-12-06

**Authors:** Ling Liu, Min Xiang, Xiangsheng Cai, Benqing Wu, Chaohong Chen, Nali Cai, Dang Ao

**Affiliations:** 1 Department of Pediatrics, Affiliated Hospital of Guangdong Medical University, Zhanjiang, 524000 Guangdong China; 2 Department of Orthopedics, Affiliated Hospital of Guangdong Medical University, Zhanjiang, 524001 China; 3 Guangzhou Cadre Health Management Center, Guangzhou Eleventh People’s Hospital, Guangzhou, 510000 Guangdong China; 4 University of the Chinese Academy of Science-Shenzhen Hospital, Shenzhen, 518000 Guangdong China

**Keywords:** White matter injury, Gut microbiota, *g_Staphylococcus*, Diffusion tension imaging, Microbiota-gut-brain axis

## Abstract

**Objective:**

The goal of this study was to comprehensively investigate the characteristics of gut microbiota dysbiosis and metabolites levels in very low or extremely low birth weight (VLBW/ELBW) infants with white matter injury (WMI).

**Methods:**

In this prospective cohort study, preterm infants with gestational age < 32 weeks and weight < 1.5 kg were investigated. Additionally, fecal samples were collected on days zero, 14d and 28d after admission to the intensive care unit. All subjects underwent brain scan via MRI and DTI at a corrected gestational age of 37 ~ 40 weeks. Based on the results of MRI examination, the VLBW/ELBW infants were divided into two groups: WMI and non-WMI. Finally, based on a multi-omics approach, we performed 16S rRNA gene sequencing, LC-MS/MS, and diffusion tension imaging to identify quantifiable and informative biomarkers for WMI.

**Result:**

We enrolled 23 patients with and 48 patients without WMI. The results of 16S RNA sequencing revealed an increase in the number of *Staphylococcus* and *Acinetobacter* species in the fecal samples of infants with WMI, as well as increasing levels of S. caprae and A._johnsonii. LEfSe analysis (LDA ≥ 4) showed that the WMI group carried an abundance of Staphylococcus species including S. caprae, members of the phyla Bacteroidota and Actinobacteriota, and *Acinetobacter* species. A total of 139 metabolic markers were significantly and differentially expressed between WMI and nWMI. KEGG pathway enrichment analysis revealed that the WMI group showed significant downregulation of 17 metabolic pathways including biosynthesis of arginine and primary bile acids. The WMI group showed delayed brain myelination, especially in the paraventricular white matter and splenium of corpus callosum. *Staphylococcus* species may affect WMI by downregulating metabolites such as cholic acid, allocholic acid, and 1,3-butadiene. Gut microbiota such as *Acinetobacter* and Bacteroidetes may alter white matter structurally by upregulating metabolites such as cinobufagin.

**Conclusion:**

Based on 16S RNA sequencing results, severe gut microbiota dysbiosis was observed in the WMI group. The results might reveal damage to potential signaling pathways of microbiota-gut-brain axis in gut microbiota. The mechanism was mediated via downregulation of the bile acid biosynthetic pathway.

**Supplementary Information:**

The online version contains supplementary material available at 10.1186/s12866-023-03103-5.

## Introduction

White matter injury (WMI) is characterized by the loss of oligodendrocyte precursor cells (OPCs), which is the main manifestation of brain injury in preterm infants, occurring in more than 50% of very low birthweight (VLBW) infants [1, 2]. The clinical manifestations of WMI in preterm infants are diverse. The motor deficits can range from mild incoordination and abnormal intonation to cerebral palsy. A recent study of the French Preterm Birth Cohort (EPIPAGE) found that 40–50% of preterm infants with gestational age (GA) less than 32 weeks showed neurodevelopmental delay at the corrected age of 2 years [3], and about 10% of VLBW manifested cerebral palsy [4, 5].

Gut microbiota comprises trillions of microorganisms. It is the largest ecosystem and the “second genome” of humans, and plays a vital role in maintaining the immune system and health [6]. However, the composition and diversity of gut microbiota are unstable and highly susceptible to food, living environment, drugs, diseases, especially antibiotics [7]. Decreased diversity of gut microbiota and dysbiosis characterized by changes in microbial type, number, ratio and location play an important role in the pathogenesis of various diseases [7, 8]. Preterm infants exhibit increased gut colonization with potentially pathogenic bacteria, decreased microbiota diversity, reduced levels of strictly anaerobic bacteria and a relatively abundance of Proteobacteria [9, 10].

In the past decade, the importance of gut microbiota in brain function including its role in neurological diseases has been increasingly recognized as a biological marker of brain development [11, 12]. The microbiota-gut-brain axis is a bidirectional pathway connecting the gastrointestinal tract and brain, integrating neural, hormonal and immune signals between the gut and the brain, thereby regulating the potential role of gut microbiota and their metabolites in brain function [11]. The colonization of gut microbiota coincides with peaks in oligodendrocyte development and brain myelination [13]. Gut microbiota play a key role in brain function and human behavior through the complex microbiota-gut-brain axis [14]. Intestinal overgrowth of *Klebsiella* was found to be highly predictive of brain injury [15]. Impaired microbiota-gut-brain axis may drive or exacerbate brain injury in preterm infants.

Diffusion tensor imaging (DTI) is the only noninvasive technique currently available to study white matter fibers, and assess the degree of myelination of white matter fibers. Conventional T1- and T2-weighted MR imaging techniques only reveal macroscopic damage and cannot quantify the extent of white matter damage [16, 17]. Fractional anisotropy (FA) and apparent diffusion coefficient (ADC) are the most common parameters of DTI, reflecting the serious damage of white matter fibers and gray matter neurons [18]. The FA value ranges from 0 to 1, and a value close to 0 indicates immature or damaged cell membrane, myelin sheath and axon of the fiber bundle, while an FA value close to 1 indicates structural integrity [19]. The ADC value is mainly determined by the water content of the tissue, and the ADC value increases in brain injury [20].

Few studies have established a direct link between gut microbiota and brain function in preterm infants in vivo. The composition of the gut microbiota associated with WMI, as well as underlying microbiological markers, have yet to be determined. The relationship between gut microbiota and brain structure and function in preterm infants is of great significance. Gut microbiota play a key role in brain development, although the specific mechanism is still unclear. Neuroimaging is a useful, non-invasive technique to elucidate the mechanisms of the microbiota-gut-brain axis underlying the relationship between gut microbiota and neuronal health. Based on the results of MRI examination, the patients were divided into WMI and non-white matter injury (nWMI) groups.

In this study, we used 16S rRNA gene sequencing, LC-MS/MS metabolite analysis and DTI to compare the DTI parameters and gut microbiota and metabolite levels between the two groups of WMI and nWMI to elucidate the microbiota-gut-brain axis.

## Materials and methods

### General information

This prospective cohort study was conducted from April 2022 to December 2022. Written informed consent of each infant’s guardian was obtained before samples were acquired for analysis. The inclusion criteria were: (1) GA at birth < 32 weeks; (2) birth weight < 1.5 kg; (3) age at admission ≤ 24 h. The exclusion criteria were: (1) death; (2) failure to complete head MRI and DTI scan during hospitalization; (3) patients with congenital chromosomal abnormalities or genetic metabolic diseases; (4) congenital brain abnormalities; (5) images that cannot be analyzed; and (6) use of microecological preparations (probiotics, prebiotics and biostime).

### Collection of stool samples

The meconium (within 24 h after birth) and the stool samples on days 14 and 28 after birth were collected, respectively. Sterile cotton swabs were used to collect fecal samples from three different locations, and each tube was about 0.2 g (the size of a soybean). The samples were immediately sealed, labeled and transferred to the refrigerator at -80℃ and frozen until genetic analysis. The meconium, day 14 and day 28 stool samples obtained from preterm infants in the WMI group were designated as WMI1, WMI14, and WMI28, respectively. The stool samples in the nWMI1 group were named nWMI1, nWMI14, and nWMI28, respectively. The specific details are presented in Table 3S.

### DNA extraction and PCR amplification

Microbial community genomic DNA was extracted from samples using the E.Z.N.A.® soil DNA Kit (Omega Bio-tek, Norcross, GA, U.S.) according to manufacturer’s instructions. The hypervariable region V3-V4 of the bacterial 16S rRNA gene were amplified with primer pairs 338 F (5’-ACTCCTACGGGAGGCAGCAG-3’) and 806R(5’-GGACTACHVGGGTWTCTAAT-3’) by an ABI GeneAmp® 9700 PCR thermocycler (ABI, CA, USA). The PCR amplification of 16S rRNA gene was performed as follows: initial denaturation at 95 ℃ for 3 min, followed by 27 cycles of denaturing at 95 ℃ for 30 s, annealing at 55 ℃ for 30 s and extension at 72 ℃for 45 s, and single extension at 72 ℃ for 10 min, and end at 4 ℃.

### Library preparation and sequencing

Purified amplicons were pooled in equimolar and paired-end sequenced on an Illumina MiSeq PE300 platform (Illumina, San Diego,USA) according to the standard protocols by Majorbio Bio-Pharm Technology Co. Ltd. (Shanghai, China). The raw reads were deposited into the NCBI Sequence Read Archive (SRA) database (BioProject: PRJNA1013131).

### Operational taxonomic unit analysis and species annotation

The taxonomy of each Operational Taxonomic Unit (OTU) representative sequence was analyzed by RDP Classifier version 2.2 against the 16S rRNA gene database (e.g. Silva v138) using confidence threshold of 0.7. Bioinformatic analysis of gut microbiota was carried out using the Majorbio Cloud platform (https://cloud.majorbio.com). Based on the OTUs information, rarefaction curves and alpha diversity indices including observed OTUs, Chao1 richness and Shannon index were calculated with Mothur v1.30.1 [21]. The similarity among the microbial communities in different samples was determined by principal coordinate analysis (PCoA) based on Bray-curtis dissimilarity using Vegan v2.5-3 package.

### Metabolite extraction and quality control sample

50 mg sample was added to a 2mL centrifuge tube and a 6 mm diameter grinding bead was added. 400 µL of extraction solution (methanol: water = 4:1 (v:v)) containing 0.02 mg/mL of internal standard (L-2-chlorophenylalanine) was used for metabolite extraction. The samples were left at -20℃ for 30 min, centrifuged for 15 min (4℃, 13,000 g), and the supernatant was transferred to the injection vial for LC-MS/MS analysis.

### LC-MS/MS analysis

The LC-MS/MS analysis of sample was conducted on a Thermo UHPLC-Q Exactive HF-X system equipped with an ACQUITY HSS T3 column (100 mm × 2.1 mm i.d., 1.8 μm; Waters, USA) at Majorbio Bio-Pharm Technology Co. Ltd. (Shanghai, China). The pretreatment of LC/MS raw data was performed by Progenesis QI software, and a three-dimensional data matrix in CSV format was exported. Internal standard peaks, as well as any known false positive peaks (including noise, column bleed, and derivatized reagent peaks), were removed from the data matrix, deredundant and peak pooled. At the same time, the metabolites were identified by searching database, and the main databases were the HMDB (http://www.hmdb.ca/), Metlin (https://metlin.scripps.edu/) and Majorbio Database. Then, the R package “ropls”(Version 1.6.2) was used to perform principal component analysis (PCA) and orthogonal least partial squares discriminant analysis (OPLS-DA), and 7-cycle interactive validation evaluating the stability of the model. The metabolites with Variable importance in the projeciton (VIP) > 1, *p* < 0.05 were determined as significantly different metabolites. Differential metabolites among two groups were mapped into their biochemical pathways through metabolic enrichment and pathway analysis based on KEGG database (http://www.genome.jp/kegg/). Python packages “scipy.stats” (https://docs.scipy.org/doc/scipy/) was used to perform enrichment analysis to obtain the most relevant biological pathways for experimental treatments.

### MRI + DTI examination

MRI scans were performed using a 3.0T GE magnetic resonance imaging machine to perform MRI scans on the head coils of all selected patients. Conventional MRI scan sequences include: T1WI axial (TR: 2849ms/TE: 30ms) and sagittal (TR: 3266ms/TE: 38ms), T2WI (TR: 5454ms/TE: 92ms) and T2WI FLAIR (TR: 8500ms/ TE: 90ms) axial imaging. The axial scanning sequence was based on the auditory canthus line, the slice thickness was 5 mm, the slice spacing was 1 mm, and the FOV = 20 × 20. The slice thickness of the sagittal scan is 5 mm, the slice distance is 0.5 mm, and the FOV = 20 × 20; the DWI and DTI scans adopt a single-shot echo planar imaging (EPI) sequence, and the b value of the DWI scan is 0,1000 s/mm2, and the FOV = 20 × 20; DTI scanning b value is 1000s/mm2, TR: 3000ms, TE: minimum, slice thickness 5 mm, slice distance 1 mm, FOV = 20 × 20, diffusion gradient direction adopts 25 directions not on the same straight line.

The degree of WMI was classified as follows: (1) mild: abnormal T1 signaling involving ≤ 3 areas, each < 2 mm damage; (2) moderate: abnormal T1 signals in > 3 areas, each > 2 mm in size, but involving < 5% of the hemisphere; and (3) severe, involving > 5% of the hemisphere [22]. According to the results of MRI examination, the patients were divided into white matter injury group (WMI) and non-white matter injury group (nWMI).

### Image analysis

The color FA map was obtained by post-processing the original DTI diffusion image with FLnctool functional software developed by GE Company in the United States. Regions of interest (ROIs) were selected on T2WI, and 8 ROI regions were measured in the frontal white matter, parietal white matter, occipital white matter, periventricular white matter, anterior and posterior limb of internal capsule, genu and splenium of corpus callosum. All ROIs were measured bilaterally symmetrically, and the size of the ROI was 15–25 mm. In order to reduce the error, each ROI was measured 3 times by two experienced radiology technicians independently.

### Statistical analysis

All statistical analyses were performed using IBM SPSS Statistics 26 and R software (Version 2.15.3). Normally distributed data were analyzed using the Student’s t test. Data that did not conform to normal distribution (such as alpha diversity, relative abundance of bacteria) were analyzed using non-parametric test (Mann-Whitney U test or Wilcoxon rank sum test). Chi-squared or Fisher’s exact test was used to identify differences between categorical variables. Linear discriminant analysis effect size (LEfSE) with an LDA cutoff of 4 was also used to identify the unique taxa or function that differentiated the groups, based on statistically significant biomarkers. Spearman correlation analysis was used to analyze the relationship between gut microbiota and metabolites and DTI parameters (FA and ADC) at different sites. Differences with *P* < 0.05 were considered significant. ^*^P < 0.05, ^**^P < 0.01, ^***^P < 0.001.

## Results

### Clinical features of WMI and nWMI groups

From April 2022 to December 2022, the Affiliated Hospital of Guangdong Medical University enrolled a total of 83 subjects who met the inclusion criteria. The study finally included 71 cases after excluding 4 deaths, 6 patients who failed to complete MRI + DTI testing, 1 case lost to treatment, and 1 case of inherited metabolic disease. Our sample finally included 23 cases of WMI and 48 cases of nWMI. The incidence of WMI in preterm infants was 32.3% (23/71). The WMI group included 12 cases (16.9%) of mild WMI, 7 cases (9.8%) of moderate WMI, and 4 cases (5.6%) of severe WMI. The incidence of moderate and severe WMI was 15%. The GA of the WMI group was 30.0 ± 1.8 weeks, while that of the nWMI group was 29.9 ± 1.7 weeks; the difference was not statistically significant. No significant differences in birth weight, sex, prenatal hormone levels, prenatal antibiotic use, APGAR score and postnatal antibiotic use were found between the two groups (*P* > 0.05), as shown in Table 1.

**Table 1:** Demographic characteristics and clinical data for the participants

Character	WMI (*n* = 23)	nWMI (*n* = 48)	*P*
Gestational age ($\overline{x}$ ± s, week)	30.0 ± 1.8	29.9 ± 1.7	0.607
Birth weight ($\overline{x}$ ± s, kg)	1.32 ± 0.21	1.30 ± 0.22	0.663
Male (n, %)	12(52.2%)	29(60.4%)	0.511
Caesarean section (n, %)	14(60.8%)	21(43.7%)	0.177
IVF-ET (n, %)	3(13%)	10(20.8%)	0.427
Asphyxia (n, %)	9(39.1%)	19(39.5%)	0.971
Hypothermia at birth (n, %)	13(56.5%)	25(52%)	0.726
SGA (n, %)	1(4.3%)	3(6.2%)	0.745
Hypoglycemia (n, %)	5(21.7%)	13(27%)	0.628
Positive pressure ventilation time (IQR, day)	10(7, 13)	6(6.3, 10.5)	0.281
Duration of antibiotic use (IQR, day)	6(5, 9.1)	5.5(4.6, 6.9)	0.188
Sepsis (n, %)	10(43.4%)	20(41.6%)	0.885
Digestive complications (n, %)	13(56.5%)	31(66.6%)	0.406
Total intestinal feeding time (IQR, day)	18.5(15, 23)	15.5(15, 19)	0.317
EUGR (n, %)	15(65.2%)	27(56.2%)	0.472
BPD (n, %)	4(17.3%)	9(18.7%)	0.89
Length of stay ($\overline{x}$ ± s,day)	50.13 ± 17.4	50.88 ± 13.3	0.391
Premature rupture of fetal membranes (n, %)	7(30.4%)	8(16.6%)	0.184
Prenatal hormones (n, %)	10(43.4%)	23(47.9%)	0.726
Prenatal antibiotic use (n, %)	12(52.1%)	18(37.5%)	0.241
GDM (n, %)	2(8.6%)	12(25%)	0.106
Preeclampsia (n, %)	2(8.6%)	10(20.8%)	0.202

*IVF-ET* In vitro fertilization-embryo transfer; *IQR *Interquartile range; *SGA *Small for gestational age; *BPD* bronchopulmonary dysplasia; *GDM* Gestational diabetes mellitus; *EUGR *Extrauterine growth retardation

### Differences in ADC and FA values between WMI and nWMI groups

**Fig. 1:**
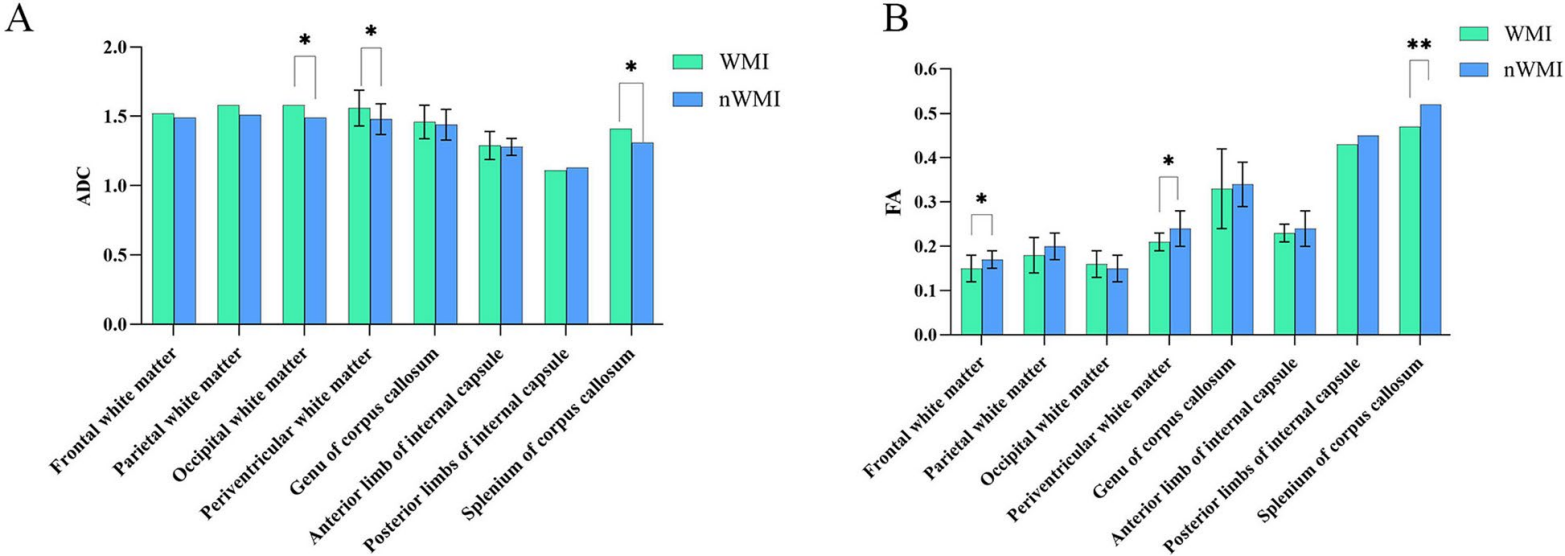
Comparison of DTI values in ROIs between WMI and nWMI group. **A** Comparison of ADC values in ROIs between WMI and nWMI group. **B** Comparison of FA values in ROIs between WMI and nWMI group. *P < 0.05, **P < 0.01

No significant difference was found in FA and ADC values of ROIs in different areas of the left and right cerebral hemispheres. Therefore, these values were averaged before comparison of the two groups. Significant differences were identified in ADC values in 3 ROIs including the occipital white matter, paraventricular white matter, and splenium of corpus callosum between the WMI and nWMI groups (Table 1s, Fig. 1A). Comparison of the FA values of ROIs in different areas of the two groups revealed significant differences in frontal white matter, paraventricular white matter, and splenium of corpus callosum (Table 2s, Fig. 1B). These results indicated that brain myelination was delayed in the WMI group, especially in the paraventricular white matter and the splenium of corpus callosum.

### Comparison of gut microbiota richness and diversity

**Fig. 2:**
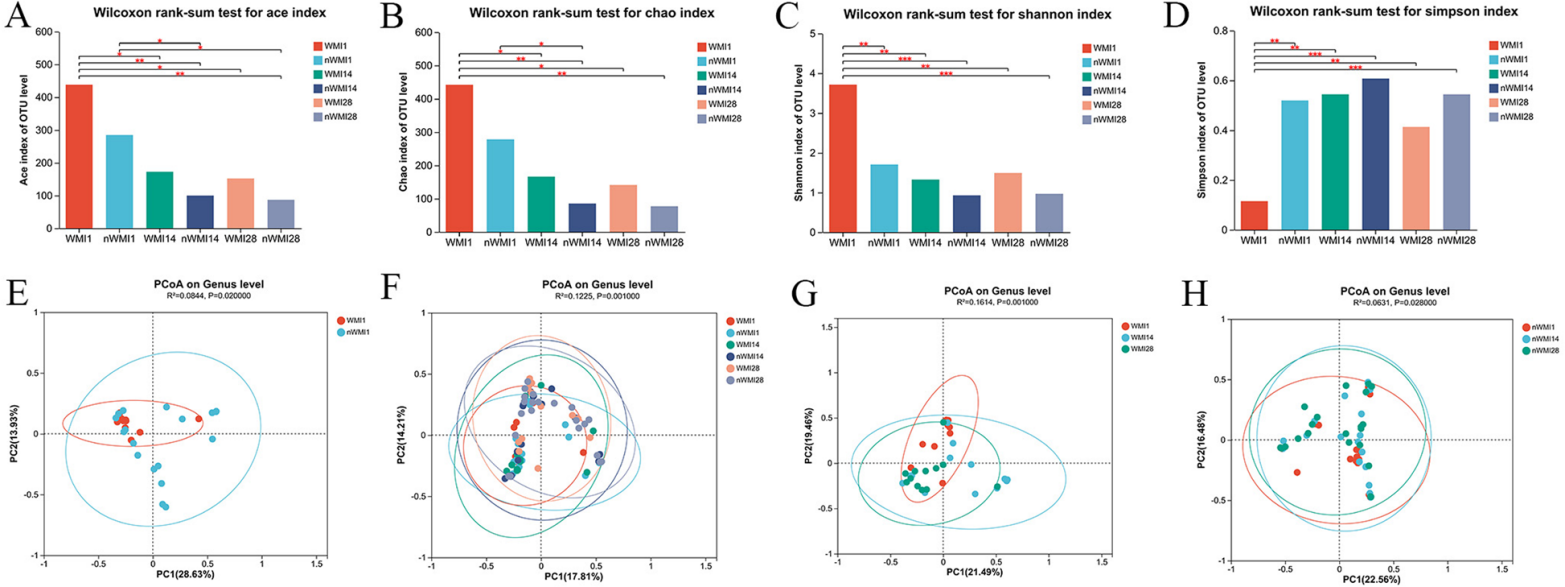
Comparison of alpha diversity and beta diversity index. (AD) Comparison of alpha diversity index, Ace (**A**), Chao (**B**), Shannon (**C**) and Simpson (**D**) index of WMI1, WMI14, WMI28, nWMI1, nWMI14, and nWMI28 at the genus level. **E** The gut microbiota composition was significantly different at the genus level between WMI1 and nWMI1(PCoA). **F** There were significant differences in the composition of gut microbiota among six groups (PCoA). **G** There were significant differences in the composition of gut microbiota at the genus level among nWMI1, nWMI14 and nWMI28 groups (PCoA). **H** There were significant differences in the composition of gut microbiota at the genus level among WMI1, WMI14 and WMI28 groups (PCoA). Differences between groups were compared using the Wilcoxon rank sum test. *P < 0.05, **P < 0.01, ***P < 0.001

The microbial community richness and community evenness were evaluated via alpha diversity analysis. The alpha diversity of preterm infants gradually decreased with age and stabilized at 4 weeks after birth (Fig. 2). As shown in Fig. 2A-C, the Ace, Chao, and Shannon indices of the WMI1 group were the highest in the WMI group, with statistically significant differences. In addition, the Shannon index of the WMI1 group was significantly higher than that of nWMI1 (*P* = 0.0035), while the Simpson index was significantly lower than that of the nWMI1 group (*P* = 0.002) (Fig. 2A-D). Significant differences were found in the diversity indices including Shannon and Simpson indices between WMI and nWMI groups, indicating that the diversity of WMI was higher than in children with nWMI (Fig. 1S).

PCoA based on Bray-Curtis distance showed that the structures of WMI1 and nWMI1 group were significantly separated at the genus level (R2 = 0.084, *P* = 0.02; Fig. 2E). Both the WMI and nWMI groups showed significant structural differences in meconium sample flora on days 14 and 28 (Fig. 2F-H). PCA analysis further revealed significant differences in overall microbial diversity between patients with WMI and nWMI controls (R2 = 0.027, *P* = 0.022; Fig. 1S). WMI samples were more dispersed compared with clustered nWMI samples, indicating the diversity in the composition of the bacterial community.

### Comparison of gut microbiota structure in feces

**Fig. 3:**
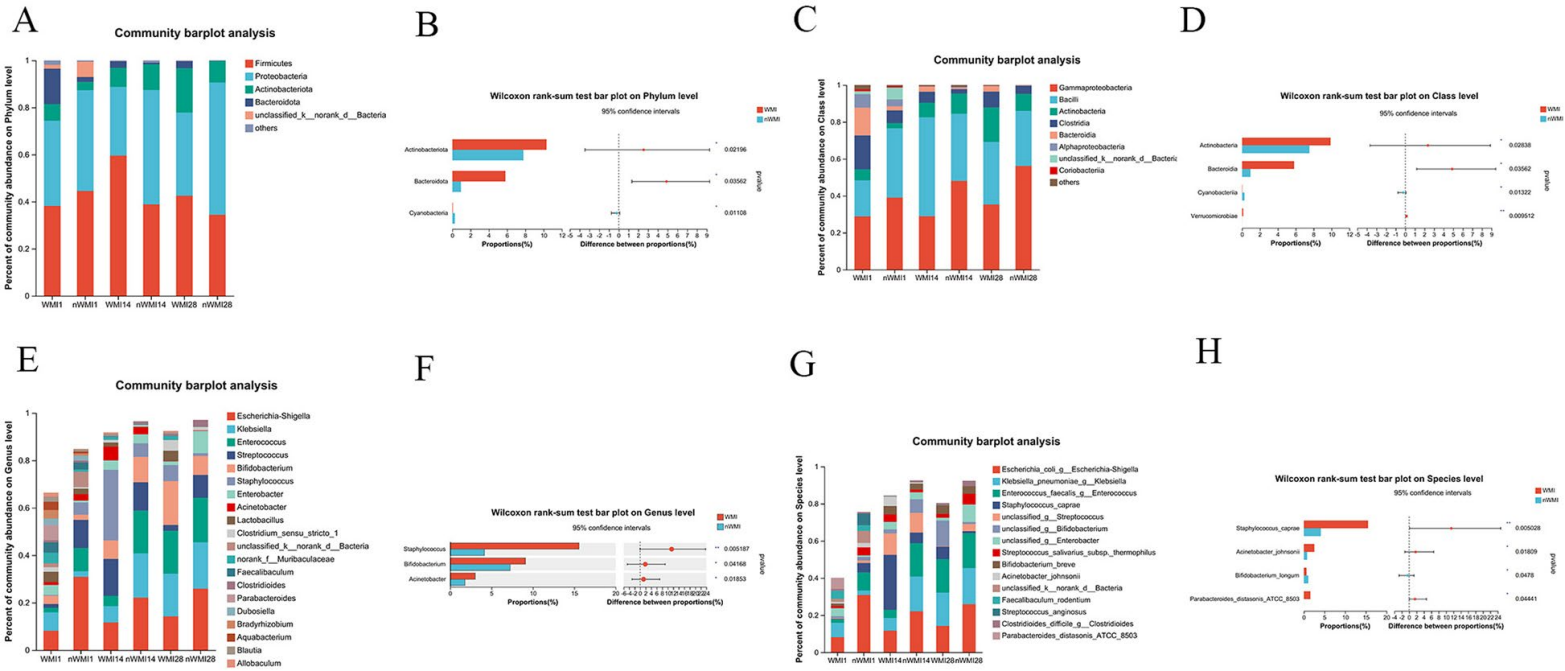
16S rRNA gene sequencing analysis. **A** The histogram shows six groups of dominant species at the phylum level. **B** Histogram showing differences in WMI and nWMI at the phylum level. **C** The histogram shows six groups of dominant species at the class level. **D** Histogram showing the difference between WMI and nWMI at the class level. **E** The histogram shows six groups of the top 15 dominant species at the genus level. **F** Histogram showing the difference between WMI and nWMI at the genus level. **G** Histogram shows six groups of the top 15 dominant species at the species level. **H** Histogram showing the difference at the species level between WMI and nWMI. *P < 0.05, **P < 0.01, ***P < 0.001

The relative abundances of OTUs in the top 10 phyla are shown (Fig. 3A). Firmicutes and Proteobacteria were the main phyla in the WMI and nWMI groups, followed by Actinobacteria and Bacteroidetes (Fig. 3A). Compared with WMI1, the relative abundance of Bacteroides, Fusobacteriota, and Verrucomicrobia was significantly lower in WMI14 and WMI28 (Kruskal-Wallis rank sum test, Fig. 2S). The results showed that the levels of Bacteroidetes, Actinobacteria and Verrucomicrobia in the WMI1 group were significantly higher than in the nWMI1 group (Wilcoxon rank test, Fig. 2S). The relative abundance of Actinobacteria and Bacteroidetes in the WMI group was higher than in the nWMI group, and the difference was significant (Wilcoxon rank sum test, *P* values, 0.0219 and 0.0356, respectively; Fig. 3B). The relative abundance of Cyanobacteria was lower than in the nWMI group.

At the class level, compared with WMI14 and WMI28, the relative abundance of Clostridia, Bacteroidia and Alphaproteobacteria in WMI1 was significantly higher than in WMI14 and WMI28 (Fig. 2S). The relative abundance of Clostridia, Bacteroides, and Actinobacteria in WMI1 was higher than in nWMI1 group based on Wilcoxon rank sum test, and the difference was statistically significant (Figs. 2S and 3C). Compared with the nWMI group, the relative abundance of Actinobacteria and Bacteroides was significantly increased in the WMI group (Fig. 3D).

According to the Wilcoxon rank sum test, compared with nWMI1, the relative abundance of *Klebsiella* and *Parabacteroides* in the WMI1 group increased significantly, and the difference was statistically significant (Figs. 2S and 3E). Compared with nWMI14, the relative abundance of *Staphylococcus* in the WMI14 group was significantly increased, and the difference was statistically significant (Figs. 2S and 3E). At the genus level, the abundance of *Staphylococcus* and *Acinetobacter* in WMI14 was significantly higher than in WMI1 and WMI28 (Figs. 2S and 3E). The relative abundances of *Staphylococcus*, *Bifidobacterium*, *Acinetobacter* and *Lactobacillus* in the WMI group were higher than in the nWMI group, and the difference was statistically significant (Fig. 3F).

Compared with nWMI1, the relative abundance of *Klebsiella pneumoniae* and *Parabacteroides distasonis*_ATCC_8503 in the WMI1 group increased significantly, and the difference was statistically significant (Figs. 2S and 3G). Compared with nWMI14, the relative abundance of *Staphylococcus caprae* in the WMI1 group increased significantly, and the difference was statistically significant (Figs. 2S and 3G). At the species level, 153 species were significantly different between the WMI and nWMI groups; 149 species were enriched in the WMI group, including *Staphylococcus caprae*, *Acinetobacter johnsonii*, and *Parabacteroides distasonis*_ATCC_8503, while 4 species were decreased, including *Bifidobacterium longum* (Fig. 3H).

### LEfSe analysis

**Fig. 4:**
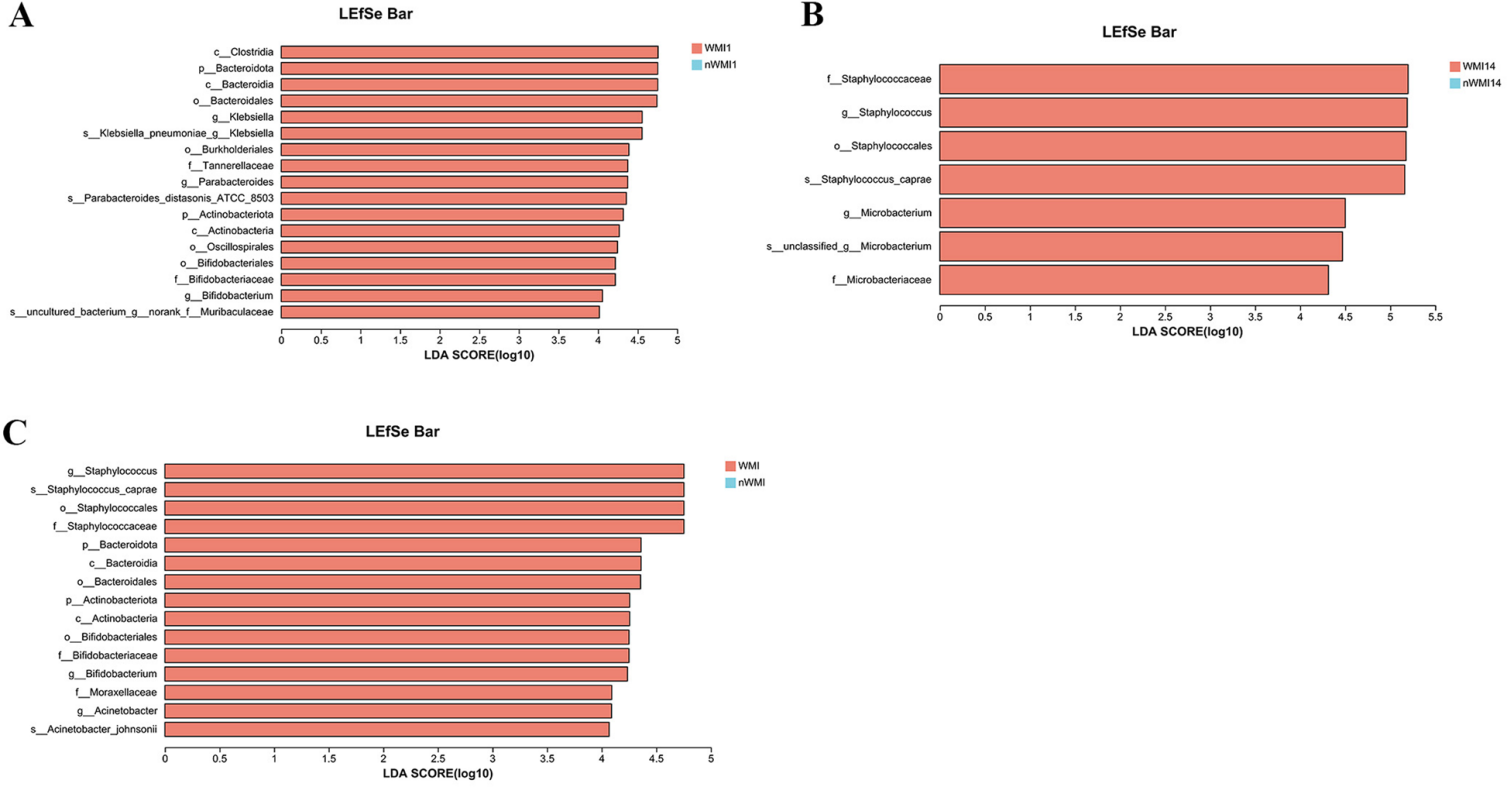
LefSe analysis. **A** Histogram of LDA values in WMI1 and nWMI1 groups. **B** Histogram of LDA values in WMI14 and nWMI14 groups. **C** Histogram of LDA values in WMI and nWMI groups

According to the LDA score, the abundance of c_*Bacteroidia*, p_*Bacteroidota*, c_Clostridia, o_Bacteroidales, g_*Klebsiella* and s_*Klebsiella pneumoniae* in WMI1 was higher than in nWMI1 (LDA > 4, Fig. 4A). After 2 weeks of birth, f_Staphylococcus, o_Staphylococcales, g_*Staphylococcus*, and s_*Staphylococcus caprae* were significantly enriched in WMI14 patient samples compared with nWMI14 based on LEfSe analysis (LDA > 4, Fig. 4B). According to the LDA score, the dominated bacteria in the WMI group were g_*Staphylococcus*, s_*Staphylococcus_caprae*, f_Staphylococcaceae, o_Staphylococcales, p_Bacteroidota, p_Actinobacteriota and g_*Acinetobacter* (Fig. 4C).

### Analysis of differential metabolites in WMI and nWMI groups

**Fig. 5:**
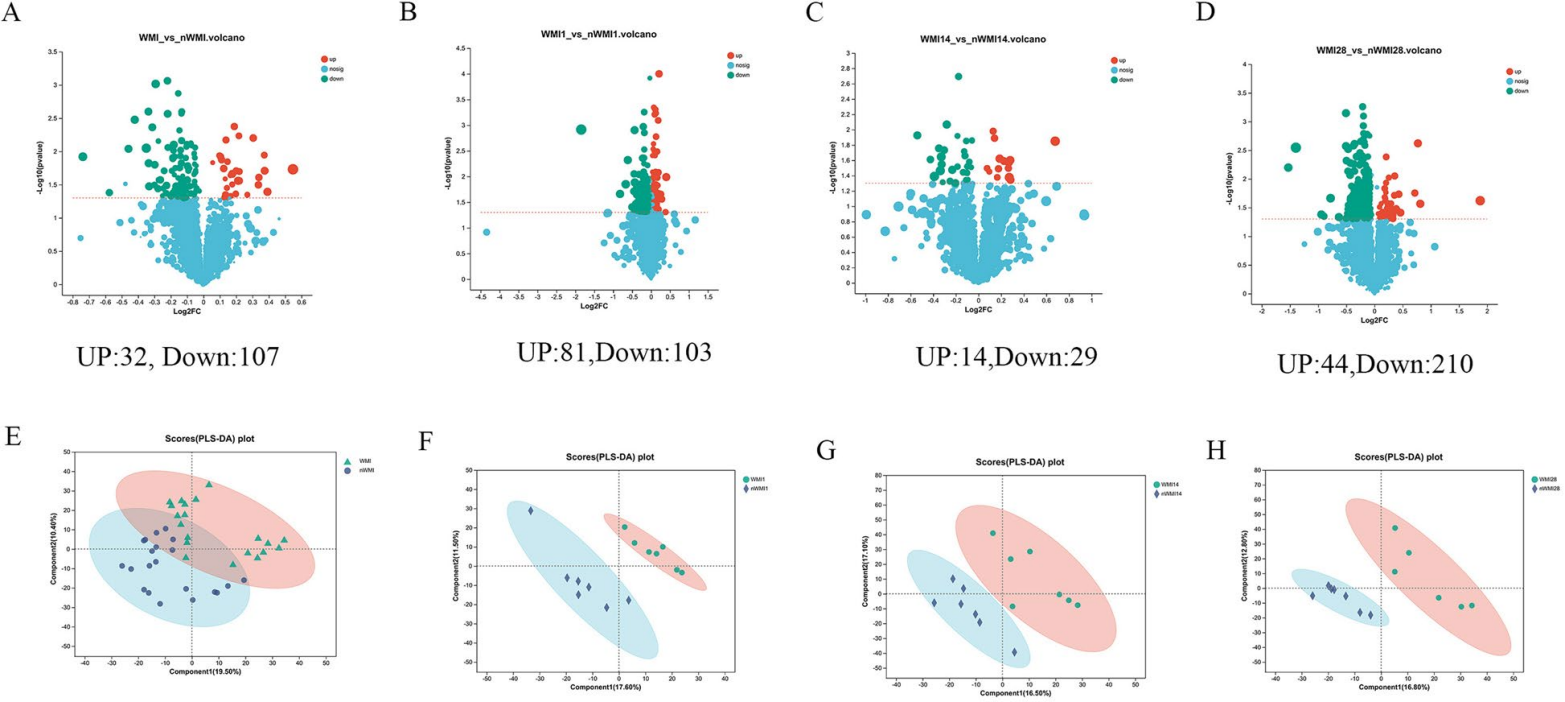
Significant difference analysis of metabolites. **A** In positive and negative ion modes, the volcano plot shows the overall distribution of significant difference metabolites in WMI and nWMI group. **B** Volcano plot showing different metabolites in the WMI1 and nWMI1 groups. **C** Volcano plot showing different metabolites in the WMI14 and nWMI14 groups. **D** Volcano plot showing significantly different metabolites in the WMI28 and nWMI28 groups. **E** PLS-DA plot of metabolite differences between WMI and nWMI group. **F** PLS-DA plot of metabolite differences between WMI1 and nWMI1 group. **G** PLS-DA plot of metabolite differences between WMI14 and nWMI14 group. **H** PLS-DA plot of metabolite differences between WMI28 and nWMI28 group

Significantly different metabolites were selected based on the variable importance in the projection (VIP) obtained from the Orthogonal Partial Least Squares Discriminant Analysis (OPLS-DA) model and the P value of the t test. Metabolites with VIP > 1 and *P* < 0.05 were considered significantly different metabolites. A total of 139 potential metabolic markers were identified between WMI and nWMI groups; 32 metabolites were significantly upregulated in the WMI group, while 107 metabolites were significantly downregulated (VIP > 1 and *P* < 0.05; Fig. 5A). A total of 184 significantly different metabolites were identified compared with the nWMI1 group, with the levels of 81 increased and 103 decreased in the WMI1 group (Fig. 5B). A total of 43 significantly different metabolites were identified, including 14 increased and 29 decreased compounds in the WMI14 group compared with the nWMI14 group (Fig. 5C). A total of 254 significantly different metabolites were identified in the WMI28 group compared with the nWMI28 group, with the levels of 44 increased and 210 decreased (Fig. 5D). The significantly upregulated metabolites included mainly lipids and lipid-like molecules, and organic compounds that contain oxygen in the WMI group. The significantly downregulated metabolites included mainly organic acids and derivatives. Clear differences were found in metabolites between the WMI and nWMI groups (Fig. 5E-H).

### Functional indicators of the faecal metabolome

**Fig. 6:**
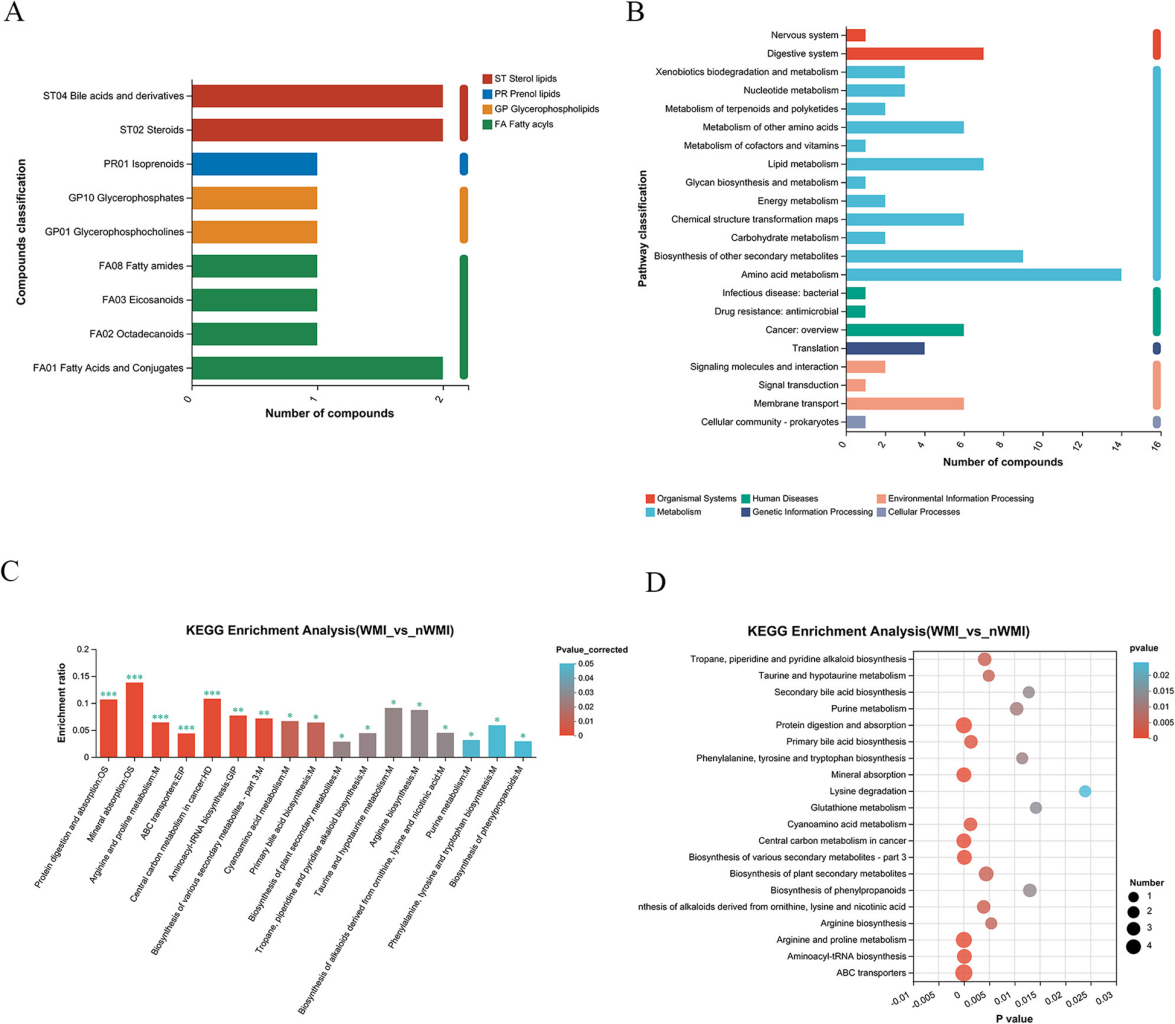
Changes in KEGG metabolic pathways and functions. **A** Compound classification (lipids) of differential metabolites in WMI and nWMI groups. **B** Major functional pathways of differential metabolites in WMI and nWMI groups. **C** KEGG metabolic pathway enrichment map (histogram) of differential metabolites in WMI and nWMI groups. **D** KEGG metabolic pathway enrichment map (bubble plot) of differential metabolites in WMI and nWMI groups

Compound classification (lipids) of differential metabolites annotated by KEGG, FA01 Fatty Acids and Conjugates, ST04 Bile acids and derivatives, and ST02 steroids in WMI and nWMI groups were the most (Fig. 6A). Among the functional pathways annotated by KEGG, Amino acid metabolism, Biosynthesis of other secondary metabolites and Lipid metabolism pathway were the most significant (Fig. 6B). KEGG enrichment analysis revealed statistically significant differences in 17 metabolic pathways between WMI and nWMI groups (adjusted *P* value < 0.05). Compared with nWMI group, taurine and hypotaurine metabolism, arginine biosynthesis, phenylalanine, tyrosine and tryptophan biosynthesis, cyanoamino acid metabolism, and primary metabolic pathways such as primary bile acid biosynthesis were downregulated in the WMI group (Fig. 6C, D). These results indicated that metabolomics and related pathways were significantly altered in WMI compared with nWMI.

### Integration of 16S rRNA genes and metabolomes

**Fig. 7:**
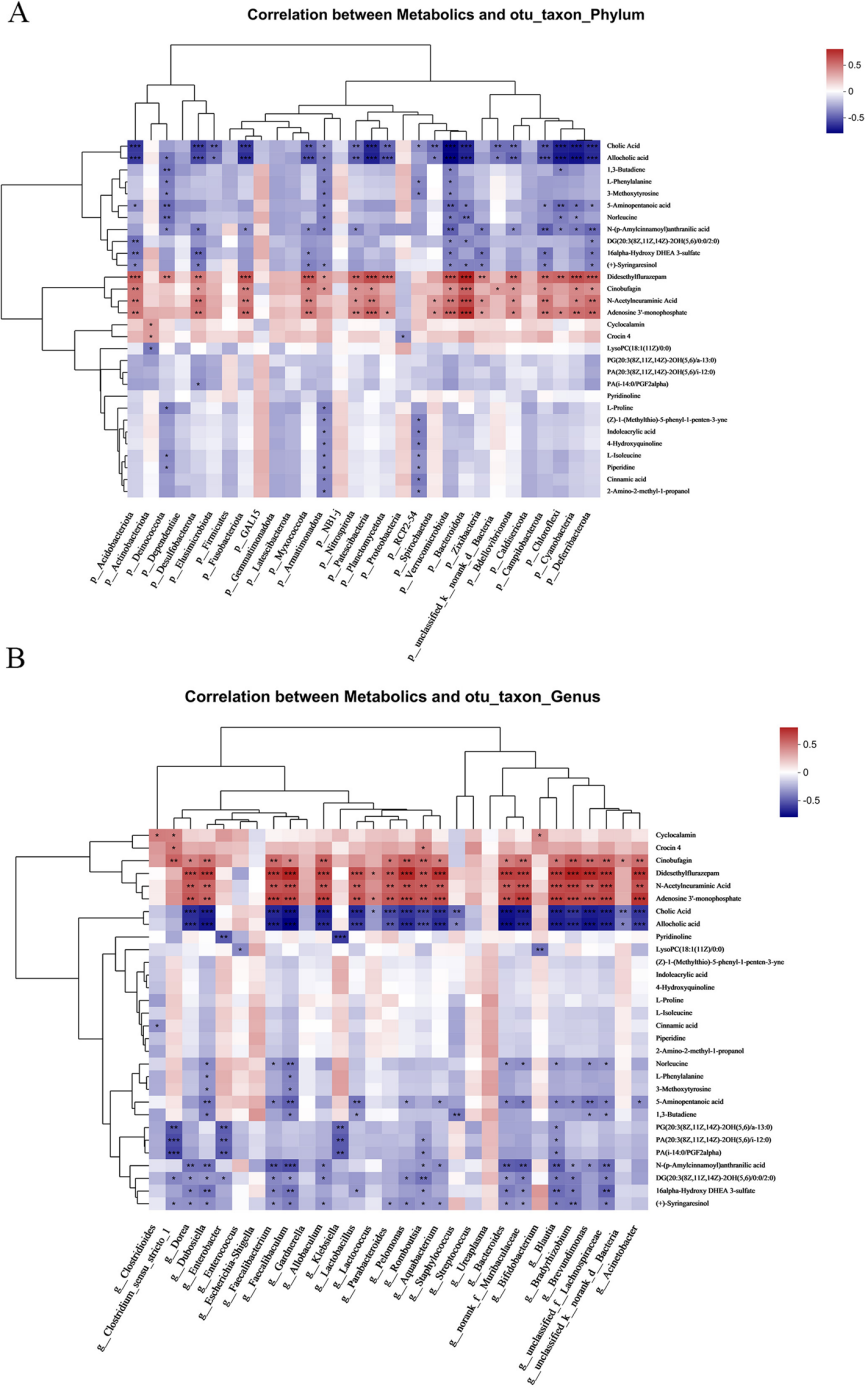
Spearman correlation analysis of gut microbiota abundance and metabolites. **A** Correlation analysis between gut microbiota and metabolites at the phylum level. **B** Correlation analysis between gut microbiota and metabolites at the genus level. *P < 0.05, **P < 0.01, ***P < 0.001

As shown in Fig. 7A, a general positive correlation was found between the WMI-characteristic Bacteroidetes and metabolites such as didesethyl flurazepam, cinobufagin, N-acetylneuraminic acid and adenosine 3’-monophosphate, and a negative correlation with metabolites such as cholic acid and allocholic acid (correlation coefficients were − 0.6135 and − 0.6253; *P* < 0.0001). Actinobacteria was positively correlated with cyclocalamin and crocin 4 (Fig. 7A).

As shown in Fig. 7B, the WMI group showed a negative correlation between *Staphylococcus* and cholic acid, allocholic acid, and 1,3-butadiene levels. The microbiota in the WMI group including *Acinetobacter* showed a positive correlation with the levels of cinobufagin, didesethyl flurazepam, N-acetylneuraminic acid, and adenosine 3’-monophosphate, and other metabolites; however, they were negatively correlated with cholic acid and allocholic acid (Fig. 7B).

The LEfSe analysis revealed that the WMI group was enriched in *Staphylococcus* species such as *S*. *caprae*, members of the phylum Bacteroidota, and *Acinetobacter* species. In the WMI group, metabolites such as didesethylflurazepam, cinobufagin, N-acetylneuraminic acid, and adenosine 3’-monophosphate were significantly upregulated, while cholic acid, allocholic acid, and 1,3-butadiene were significantly downregulated. Notably, *Staphylococcus* may affect WMI by downregulating metabolites such as cholic acid, allocholic acid, and 1,3-butadiene. However, members of phylum Bacteroidota and *Acinetobacter* species affect WMI by upregulating the levels of didesethylflurazepam, cinobufagin, N-acetylneuraminic acid, and adenosine 3’-monophosphate, while downregulating metabolites such as cholic acid and allocholic acid. The results suggested that patients with WMI carry a significantly dysregulated gut microbiota, which may lead to marked alterations in metabolomics.

### Correlation between DTI values and gut microbiota

**Fig. 8:**
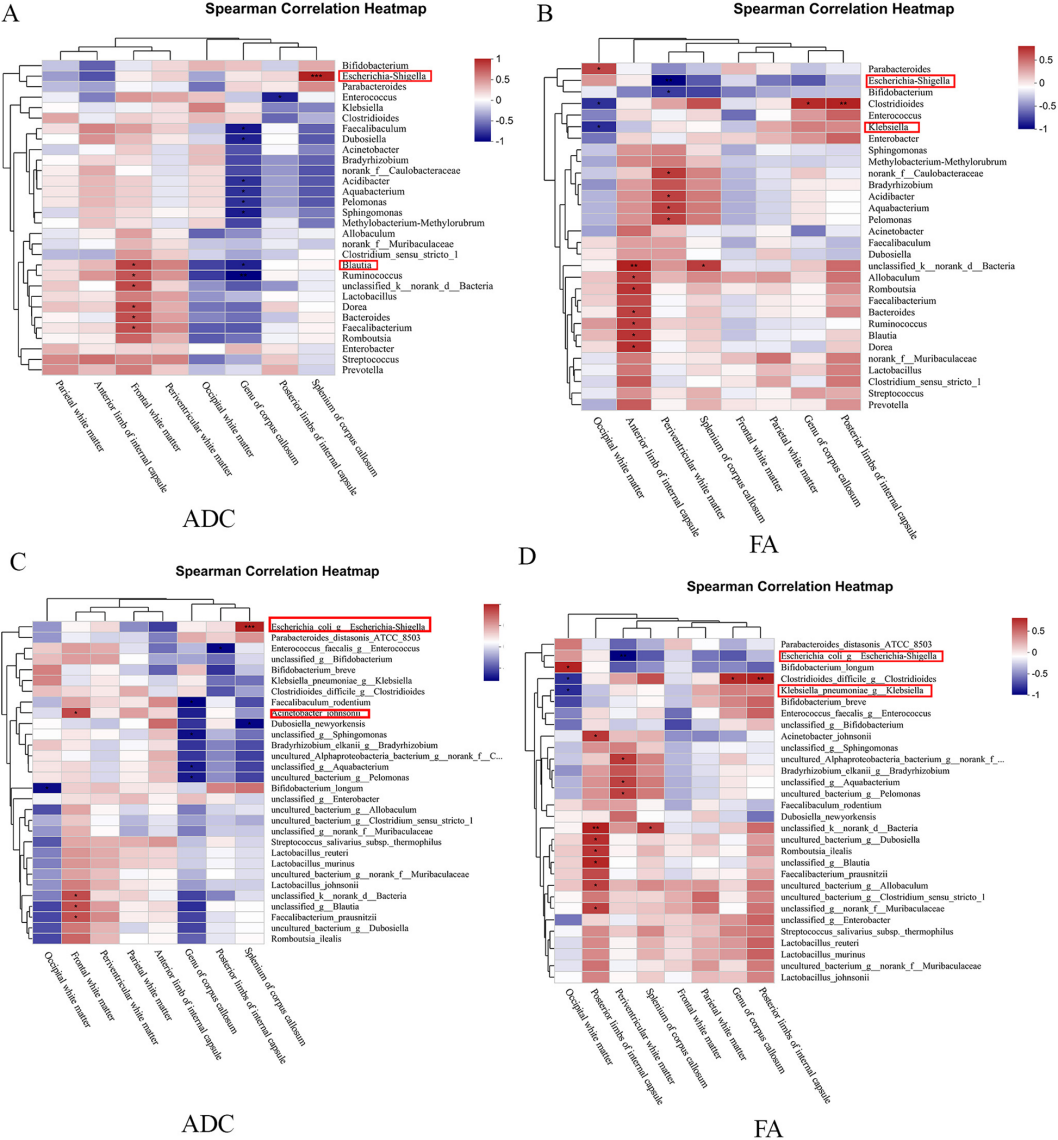
Correlation analysis between ADC and FA values and differential gut microbiota. **A** At the genus level, the heatmap of the correlation between ADC value and gut microbiota. **B** At the genus level, the heatmap of the correlation between the FA value and gut microbiota. **C** At the species level, the heatmap of the correlation between the ADC value and gut microbiota. **D** At the species level, the heatmap of the correlation between the FA value and gut microbiota. *P < 0.05, **P < 0.01

The results showed that *Escherichia-Shigella* was positively correlated with the ADC value of splenium of corpus callosum (Fig. 8A), while *Blautia* species were positively correlated with the ADC value of frontal white matter (Fig. 8A). *Escherichia-Shigella* was negatively correlated with the FA value of periventricular white matter (Fig. 8B), while *Klebsiella* was negatively correlated with the FA value of occipital white matter (Fig. 8B). Therefore, *Escherichia-Shigella* may be related to the splenum of corpus callosum and periventricular white matter; *Blautia* may be related to brain damage associated with frontal white matter; and*_Klebsiella* is related to brain damage involving occipital white matter.

The heatmap showed that *Bifidobacterium longum* was negatively correlated with occipital white matter ADC value (Fig. 8C). *K*._*pneumoniae* was negatively correlated with the FA value of occipital white matter (Fig. 8D). Therefore, *K.*_*pneumoniae* is related to brain damage of occipital white matter. *B*. *longum* may have a protective effect on occipital white matter, suggesting a novel therapeutic role of probiotics in WMI.

### Correlation between DTI values and differential metabolites

**Fig. 9:**
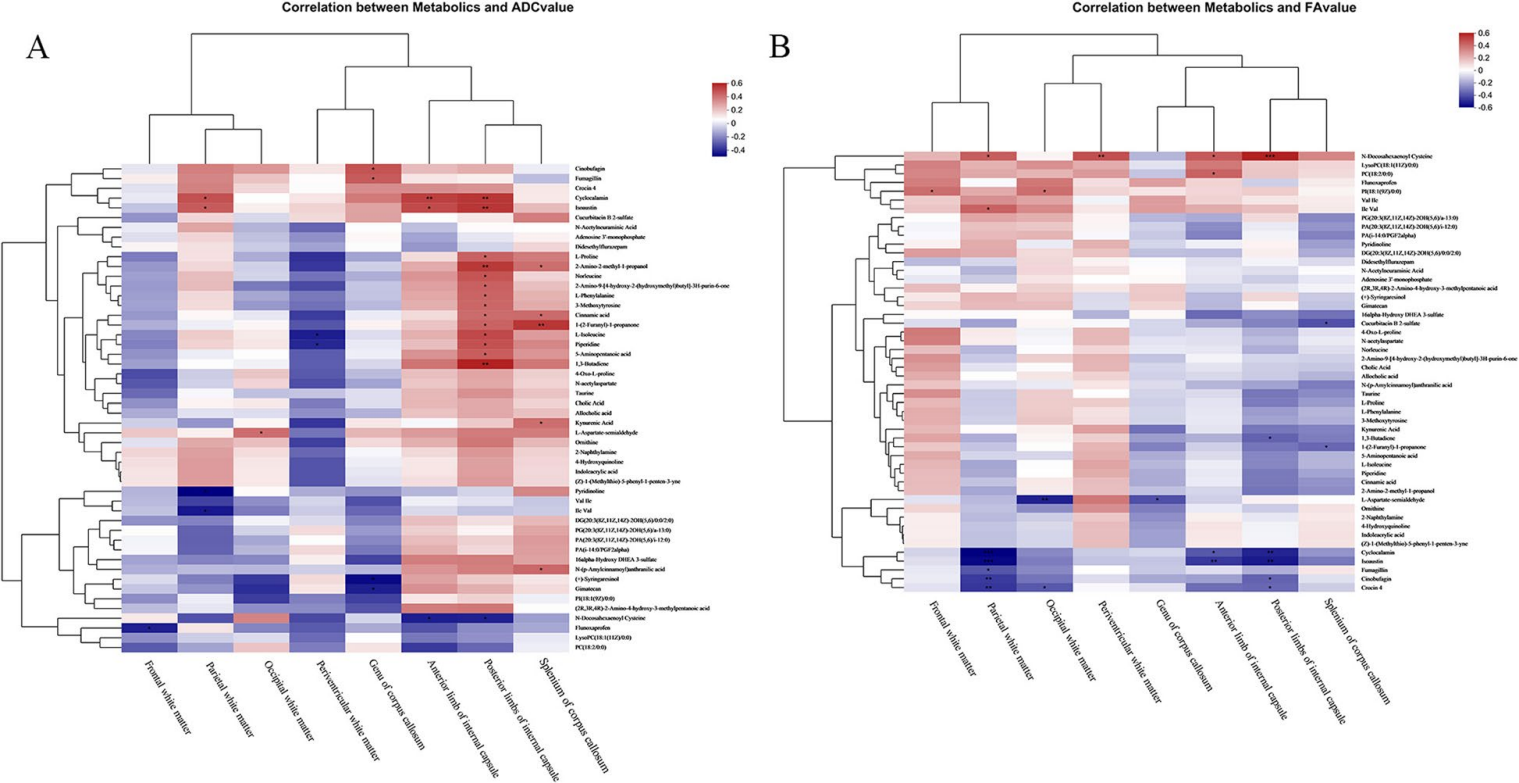
Correlation between intestinal metabolites and DTI values. **A** Correlation between intestinal metabolites and white matter ADC values. **B** Correlation of gut metabolites with white matter FA values. *P < 0.05, **P < 0.01

Based on Spearman correlation analysis, we found that cinobufagin and fumagillin were positively but weakly correlated with the ADC of genu of corpus callosum (Fig. 9A). Cyclocalamin was positively correlated with the ADC values of parietal white matter, anterior limb of internal capsule, and posterior limbs of internal capsule (Fig. 9A). However, N-docosahexaenoyl cysteine was negatively correlated with the ADC of anterior limb of internal capsule and ADC of posterior limbs of internal capsule (Fig. 9A).

Cyclocalamin and parietal white matter FA, anterior limb of internal capsule, and posterior limbs of internal capsule showed a negative correlation (Fig. 9B). Fumagillin, cinobufagin, and crocin 4 were negatively correlated with parietal white matter FA. N-Docosahexaenoyl cysteine was positively correlated with the FA values of parietal white matter, periventricular white matter, anterior limb of internal capsule, and posterior limbs of internal capsule.

Cinobufagin, fumagillin, cyclocalamin, isoaustin, crocin 4 and other metabolites were positively correlated with ADC values in different regions of white matter in the brain, and negatively correlated with FA values, which suggest brain damage. By contrast, N-docosahexaenoyl cysteine was negatively correlated with ADC values of different regions of white matter in the brain but positively correlated with FA, suggesting protective effects. Based on the correlation between the abundance of gut microbiota and metabolites, the characteristic *Acinetobacter* species and Bacteroidetes of WMI group were positively correlated with metabolites such as cinobufagin. Therefore, gut microbiota such as *Acinetobacter* and Bacteroidetes may affect white matter structure by upregulating the levels of metabolites such as cinobufagin.

## Discussion

This is the first study to investigate the role of white matter structure, gut microbiota and metabolites in VLBW/ELBW infants and the potential relationship between these factors. In this study, by integrating 16S rRNA gene sequencing and LC-MS/MS metabolomics, we found not only significant differential expression of metabolites between WMI and nWMI groups, but a significant correlation between the different flora and metabolites possibly involved in the pathogenesis of WMI. This prospective cohort study revealed that WMI was characterized by significant gut microbiota dysbiosis, and provided new insights into the status of gut microbiota in WMI and WMI-related biomarker levels.

Our study found that compared with the nWMI group, the abundance of Actinobacteria and Bacteroidetes in the WMI group was significantly increased. Bacteroidetes, the largest phylum of gram-negative bacteria in the GI microbiota, are beneficial to the host when confined to the GI tract, but have the potential to secrete pro-inflammatory neurotoxins, including lipopolysaccharide (LPS) and other toxic proteolytic peptides, thereby damaging the nervous system [23]. Zhanfeng et al(2022). reported that Bacteroidetes were predominant in the sleep disorder group [24]. It was also found that the Bacteroidetes secrete LPS, which triggers an inflammatory response and aggravates the progression of diabetes [25].

This study found that the relative abundance of *Staphylococcus*, *Bifidobacterium*, *Acinetobacter* and *lactobacillus* in the WMI group was higher than in the nWMI group. You, W et al. (2022). found that traumatic brain injury induced gut microbiota dysbiosis and changed the distribution of bile acids, suggesting the role of *Staphylococcus* in altered bile acid metabolism [26]. Compared with nWMI14, the relative abundance of *Staphylococcus* was significantly increased in the WMI14 group. The WMI group was colonized with abnormal *Staphylococcus* within 14 days of life. In the nWMI group, the abundance of *Staphylococcus* decreased by week 2 after birth; while the abundance of *Staphylococcus* in the WMI group increased significantly at week 2 after birth, persisting until 28 days after birth. The WMI group showed an abnormal increase in the abundance of *Staphylococcus* within 1 month after birth. Our study also found that the abundance of *Acinetobacter* was significantly and abnormally increased in the WMI group on day 14 after birth, while the abundance of *Acinetobacter* in the nWMI group did not appear to be abnormally increased. Cekanaviciute, E et al. (2017). reported that the abundance of *Acinetobacter* was significantly increased in patients with multiple sclerosis, which can induce pro-inflammatory response [27]. Inhibition of early abnormal colonization of *Staphylococcus* and *Acinetobacter* in preterm infants may facilitate the prevention and treatment of WMI. Our study also found that the WMI group was enriched in *Lactobacillus* and *Bifidobacterium*. Although *Lactobacillus* and *Bifidobacterium* represent probiotics, many studies have shown that the relative abundance of *Lactobacillus* increased in the gut microbiota of patients with high-fat diet and diabetes, and *Bifidobacterium* was detected in different infections [28]. The relative abundance of *Klebsiella* and *Parabacteroides* in the meconium of WMI group was significantly higher than in the nWMI group. Patients with extrauterine growth retardation carry an abundance of *Parabacteroides*, which can affect the storage of sugar in muscle, liver and fat [9]. Fecal samples derived from preterm infants were significantly enriched with *Klebsiella* within 2 weeks of life, whereas full-term infants showed an increase in *Klebsiella* after 6 months of life [29]. Notably, *Klebsiella* is associated with pro-inflammatory response, and overgrowth of *Klebsiella* in the gastrointestinal tract is highly predictive of brain injury [15]. The relative abundance of *Klebsiella* in the meconium of the WMI group was significantly higher than in the nWMI group, suggesting possible brain damage early in life. Based on LefSe analysis, the WMI group was enriched in a large number of *Staphylococcus* species, suggesting a potential role in the early diagnosis of WMI.

At the species level, compared with the nWMI group, the abundance of *S*. *caprae* was significantly higher in the WMI group, while the abundance of *B*. *longum* was significantly decreased. *S*. *caprae* is closely related to *S*. *epidermidis* and *Streptococcus capitis* at the species level, especially in the ability to form biofilms, which may increase the virulence of *S*._*caprae* [30]. *B*. *longum* and *B*. *longum* subspecies were found to exhibit protective effects. Studies have found that the probiotic *B*. *longum* NCC3001 reduced the excitability of enteric neurons and normalized the levels of hippocampal brain-derived neurotrophic factor, resulting in anti-anxiety effects [31]. Intervention with *B*. *longum* R0175, *Lactobacillus helveticus* R0052 and *L*. *plantarum* R1012 regulated brain activity and functional connectivity in emotional and stress response areas [32]. During early life, a critical period of brain growth and development, nutritional interventions (such as probiotics, psychobiologics, prebiotics, synbiotics and maternal nutrition) may have a favorable effect on gut-brain signaling, and possibly contribute to brain maturation [33]. In this study, the abundance of *B. longum* in the nWMI group increased significantly, but whether *B*. *longum* plays a protective role in the pathogenesis of WMI requires further investigation. Compared with nWMI1, the relative abundance of *K. pneumoniae* was significantly increased in the WMI1 group. The increased abundance of *K*. *pneumoniae* was associated with the risk of NEC in preterm infants [34].

Gut microbiota and metabolites not only play a role in maintaining the homeostasis of the gastrointestinal tract, but also regulate remote organs such as the brain [6]. Clinical evidence suggests that gut microbiota dysbiosis is a key predisposing factor for neuropsychiatric disorders such as Alzheimer’s disease, autism and major depressive disorder [35]. Gut microbiota could secrete large amounts of amyloid, LPS and other exudates to induce pro-inflammatory cytokines in the brain, and participate in the pathogenesis of Alzheimer’s disease [28]. Some gut microbial metabolites such as LPS, Short-chain fatty acid (SCFAs), trimethylamine and vitamins can directly affect brain neurons or stimulate the immune and endocrine systems [36]. Our results found that the gut microbiota of WMI group differed from nWMI group not only in structure and composition but also in function, with significant differences in fecal microbial metabolites between the WMI and nWMI groups. Our study also found 139 metabolites with the most significant differences between the two groups, including 32 metabolites, which were significantly upregulated and 107 metabolites significantly downregulated in the WMI group (VIP > 1 and *P* < 0.05). Compared with the nWMI group, the WMI group showed significant downregulation in metabolite pathways of taurine and hypotaurine, arginine biosynthesis, phenylalanine and primary bile acid biosynthesis. Metabolites and related functional pathways were significantly altered in patients with WMI compared with those of nWMI.

This study is the first to identify several differential metabolites that may be associated with WMI. The characteristic Bacteroidetes of WMI were generally positively correlated with metabolites such as didesethylflurazepam, cinobufagin, N-acetylneuraminic acid, and adenosine 3’-monophosphate, but they were negatively correlated with metabolites such as cholic Acid and allocholic acid. The characteristic *Acinetobacter* species of WMI group were positively correlated with metabolites such as cinobufagin, didesethylflurazepam, N-acetylneuraminic acid and adenosine 3’-monophosphate, whereas they were negatively correlated with metabolites such as cholic acid and allocholic acid. The characteristic *Staphylococcus* species of the WMI group were negatively correlated with metabolites such as cholic acid and allocholic acid. Didesethylflurazepam is a benzodiazepine. Cinobufagin is a steroid lactone. N-Acetylneuraminic acid participates in amino sugar and nucleotide sugar metabolism. Adenosine 3’-monophosphate participates in purine metabolism. Cholic acid is a bile acid and participates in primary bile acid biosynthesis. Allocholic acid is also a bile acid and participates in the synthesis of secondary bile acids. Metabolites such as didesethylflurazepam, cinobufagin, N-acetylneuraminic acid, and adenosine 3’-monophosphate were significantly upregulated in the WMI group, while cholic acid and allocholic acid were significantly downregulated. KEGG pathway analysis also showed that biosynthesis of primary and secondary bile acids in the WMI group was significantly downregulated. The main function of bile acids is to promote intestinal absorption of lipids and fat-soluble vitamins in the diet. SCFAs and bile acids also affect host energy production, and secondary bile acids help regulate innate immunity, insulin sensitivity and host metabolic pathways (including carbohydrate and lipid metabolism). In addition, secondary bile acids maintain gut microbiota balance by modulating the innate immune response of the gut [37]. Bile acids reduce the expression of pro-inflammatory cytokines in monocytes, macrophages and dendritic cells [38]. Notably, bile acids improve glucose tolerance in mice by regulating the fibroblast growth factor receptors on AGRP/NPY neurons in the hypothalamus of obese mice [39]. Shanshan Xie [40] reported that cinobufagin regulated the inflammatory phenotype of dendritic cells and triggered innate immune response. Chenze Li et al(2021). found that plasma N-scetylneuraminic acid was associated with poor clinical prognosis in patients with heart failure [41]. WMI-characteristic Bacteroidetes and *Acinetobacter* significantly increased metabolites related to purine metabolism, amino sugar and nucleotide sugar metabolism, and significantly inhibited metabolites of bile acid biosynthesis. *Staphylococcus* may alter bile acid biosynthesis, and thereby affect WMI by downregulating metabolites such as cholic acid and allocholic acid. Further, Bacteroidota and *Acinetobacter* play a role in WMI by upregulating the levels of didesethylflurazepam, cinobufagin, N-acetylneuraminic acid, and adenosine 3’-monophosphate, and downregulating cholic acid and allocholic acid levels. These results suggested that Bacteroides, *Acinetobacter* and *Staphylococcus* and related fecal microbial metabolites may regulate the microbe-gut-brain axis during the pathogenesis of WMI.

Increased ADC and decreased FA values in the periventricular white matter and splenium of corpus callosum may represent early markers of WMI in preterm infants. This study also showed that the incidence of WMI in VLBW/ELBW infants was 32.3%, and the incidence of moderate-to-severe WMI was 15.4%. Although the incidence of moderate-to-severe WMI was consistent with the study of Dawn Gano [42], birth weight and GA was lower in our study. This study investigated the potential link between gut microbiota, metabolites and white matter structure in VLBW/ELBW infants.The link between the relative abundance of gut microbiota and brain white matter structure is complex. The heatmap showed that *B*. *longum* was negatively correlated with occipital white matter ADC, suggesting a protective effect. This finding may provide a new therapeutic target for probiotics in the treatment of WMI. Our study found that cinobufagin, fumagillin, cyclocalamin, isoaustin, crocin 4 and other metabolites were positively correlated with ADC values in different regions of white matter of the brain, and negatively correlated with FA values, which may lead to brain damage. However, N-docosahexaenoyl cysteine was negatively correlated with ADC values in different regions of white matter in the brain, and the positive correlation with FA value may have a protective effect on the brain. These results suggest the need for further investigations. Ze-Jun Wang [43] reported that high concentrations of cinobufagin have serious side effects on the central nervous system, including epilepsy and coma in patients. Gut microbiota such as *Acinetobacter* and Bacteroidetes may affect the structure of white matter by altering metabolites such as cinobufagin.

The current study has several limitations. First, the sample size was relatively small, suggesting the need for studies with larger sample size. Based on current evidence, it has not possible to establish a causal relationship between alterations in the gut microbiota and metabolome and the pathogenesis of WMI, a limitation shared by observational studies. Although the precise mechanisms of gut microbiota affecting the brain are unknown, our findings suggest key signaling pathways and molecules involved in gut-brain interactions in WMI. Second, we only collected fecal samples three times, which limited the investigation of the bacterial colonization time in preterm infants. Due to the impact of the COVID-19 pandemic, the study subjects could not successfully complete the follow-up. Finally, our study lacks basic data and follow-up studies involving animal models are needed to elucidate the mechanisms outlined in this study to screen specific metabolites and pathways.

## Conclusion

In summary, these results suggest severe gut microbiota dysbiosis in patients with WMI. A few WMI-characteristic groups, such as Bacteroidetes, *Staphylococcus* and *Acinetobacter* are strongly associated with altered fecal microbial metabolites, possibly damaging the white matter in the brain by dow-regulating the bile acid biosynthesis pathway.

### Supplementary Information

**Additional file 1.**

** Additional file 2.**

** Additional file 3.**

** Additional file 4.**

** Additional file 5.**

## Data Availability

The amplicon sequencing data are available in the NCBI Sequence Read Archive (SRA) database (BioProject: PRJNA1013131). The detail data and materials available please see https://www.ncbi.nlm.nih.gov/bioproject/PRJNA1013131.
