# Supplementary figures and images for: Multi-omics analyses of gut microbiota via 16S rRNA gene sequencing, LC-MS/MS and diffusion tension imaging reveal aberrant microbiota-gut-brain axis in very low or extremely low birth weight infants with white matter injury
Source: BMC Microbiol. 2023 Dec 6;23:387. doi: 10.1186/s12866-023-03103-5 (PMC10699022; doi:10.1186/s12866-023-03103-5)

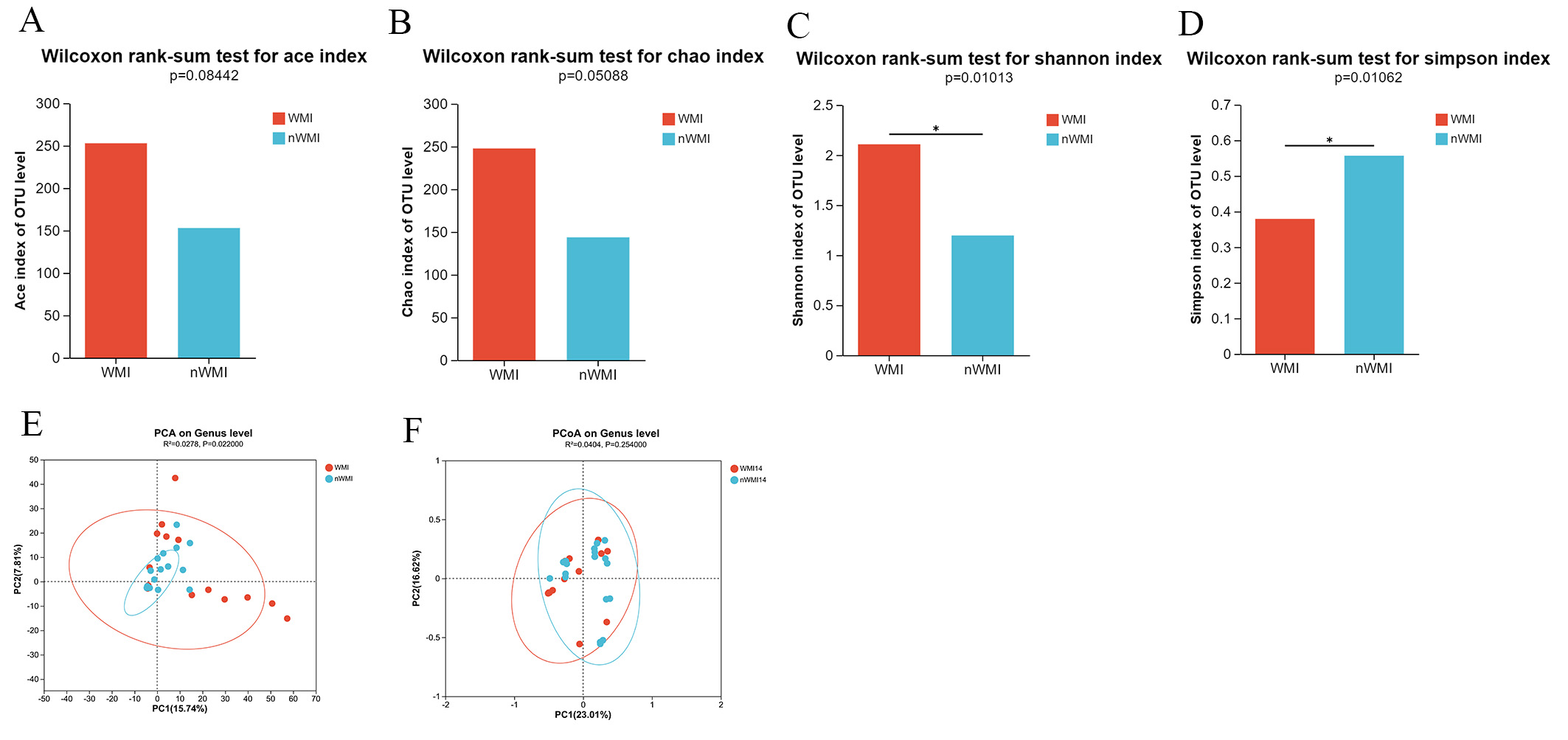

Supplement: Supplementary file 1 — Additional file 1. [file 12866_2023_3103_MOESM1_ESM.jpg]

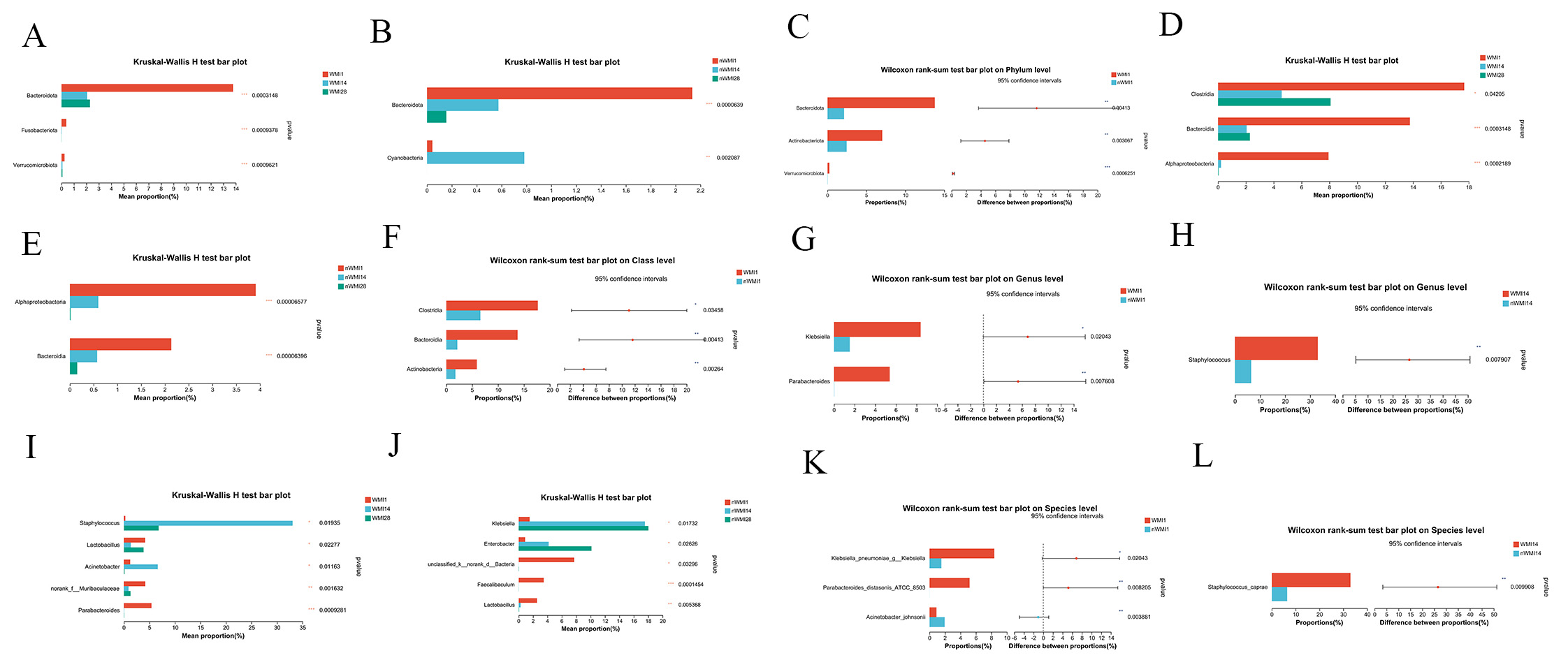

Supplement: Supplementary file 2 — Additional file 2. [file 12866_2023_3103_MOESM2_ESM.jpg]
