# Supplementary material for: Multi-omics analyses of gut microbiota via 16S rRNA gene sequencing, LC-MS/MS and diffusion tension imaging reveal aberrant microbiota-gut-brain axis in very low or extremely low birth weight infants with white matter injury
Source: BMC Microbiol. 2023 Dec 6;23:387. doi: 10.1186/s12866-023-03103-5 (PMC10699022; doi:10.1186/s12866-023-03103-5)
Supplement: Supplementary file 3 — Additional file 3. [file 12866_2023_3103_MOESM3_ESM.doc]

**Table 1s Comparison of ADC values in ROIs between WMI group and nWMI group.**

|  | WMI  （23） | nWMI  （48） | *P* |
| --- | --- | --- | --- |
| Frontal white matter | 1.52（1.44,1.59） | 1.49（1.43,1.56） | 0.337 |
| Parietal white matter | 1.58（1.46,1.66） | 1.51（1.46,1.59） | 0.248 |
| Occipital white matter | 1.58（1.49,1.65） | 1.49（1.42,1.59） | 0.016^*^ |
| Periventricular white matter | 1.56±0.13 | 1.48±0.11 | 0.032^*^ |
| Genu of corpus callosum | 1.46±0.12 | 1.44±0.11 | 0.468 |
| Anterior limb of internal capsule | 1.29±0.1 | 1.28±0.06 | 0.843 |
| Posterior limbs of internal capsule | 1.11（1.08,1.13） | 1.13（1.1,1.16） | 0.233 |
| Splenium of corpus callosum | 1.41（1.29,1.57） | 1.31（1.23,1.38） | 0.007^**^ |

ROI: Region of interest; ADC: Apparent diffusion coefficient. ^*^*P*＜0.05，^**^*P*＜0.01。
