# Supplementary material for: Multi-omics analyses of gut microbiota via 16S rRNA gene sequencing, LC-MS/MS and diffusion tension imaging reveal aberrant microbiota-gut-brain axis in very low or extremely low birth weight infants with white matter injury
Source: BMC Microbiol. 2023 Dec 6;23:387. doi: 10.1186/s12866-023-03103-5 (PMC10699022; doi:10.1186/s12866-023-03103-5)
Supplement: Supplementary file 4 — Additional file 4. [file 12866_2023_3103_MOESM4_ESM.doc]

**Table 2s Comparison of FA values in ROIs between WMI group and nWMI group.**

|  | WMI  （23） | nWMI  （48） | *P* |
| --- | --- | --- | --- |
| Frontal white matter | 0.15±0.03 | 0.17±0.02 | 0.013^*^ |
| Parietal white matter | 0.18±0.04 | 0.2±0.03 | 0.292 |
| Occipital white matter | 0.16±0.03 | 0.15±0.03 | 0.304 |
| Periventricular white matter | 0.21±0.02 | 0.24±0.04 | 0.039^*^ |
| Genu of corpus callosum | 0.33±0.09 | 0.34±0.05 | 0.678 |
| Anterior limb of internal capsule | 0.23±0.02 | 0.24±0.04 | 0.321 |
| Posterior limbs of internal capsule | 0.43（0.38,0.47） | 0.45（0.41,0.47） | 0.16 |
| Splenium of corpus callosum | 0.47（0.29,0.47） | 0.52（0.43,0.52） | 0.005^**^ |
