# Supplementary material for: Multi-omics analyses of gut microbiota via 16S rRNA gene sequencing, LC-MS/MS and diffusion tension imaging reveal aberrant microbiota-gut-brain axis in very low or extremely low birth weight infants with white matter injury
Source: BMC Microbiol. 2023 Dec 6;23:387. doi: 10.1186/s12866-023-03103-5 (PMC10699022; doi:10.1186/s12866-023-03103-5)
Supplement: Supplementary file 5 — Additional file 5. [file 12866_2023_3103_MOESM5_ESM.doc]

**Table 3s Sample and group information table**

|  | Group | Sequencing |
| --- | --- | --- |
| WMI | WMI1 | 14 |
|  | WMI14 | 17 |
|  | WMI28 | 16 |
| nWMI | nWMI1 | 35 |
|  | nWMI14 | 39 |
|  | nWMI28 | 38 |
| Total 159 | | |
